# A Systematic Identification of RNA-Binding Proteins (RBPs) Driving Aberrant Splicing in Cancer

**DOI:** 10.3390/biomedicines12112592

**Published:** 2024-11-13

**Authors:** Cesar Lobato-Fernandez, Marian Gimeno, Ane San Martín, Ana Anorbe, Angel Rubio, Juan A. Ferrer-Bonsoms

**Affiliations:** Departamento de Ingeniería Biomédica y Ciencias, TECNUN, Universidad de Navarra, 20009 San Sebastián, Spain; clobatofern@unav.es (C.L.-F.);

**Keywords:** alternative splicing, RNA-Seq, TCGA, RBPs

## Abstract

Background: Alternative Splicing (AS) is a post-transcriptional process that allows a single RNA to produce different mRNA variants and, in some cases, multiple proteins. Various processes, many yet to be discovered, regulate AS. This study focuses on regulation by RNA-binding proteins (RBPs), which are not only crucial for splicing regulation but also linked to cancer prognosis and are emerging as therapeutic targets for cancer treatment. CLIP-seq experiments help identify where RBPs bind on nascent transcripts, potentially revealing changes in splicing status that suggest causal relationships. Selecting specific RBPs for CLIP-seq experiments is often driven by a priori hypotheses. Results: We developed an algorithm to detect RBPs likely related to splicing changes between conditions by integrating several CLIP-seq databases and a differential splicing detection algorithm. This work refines a previous study by improving splicing event prediction, testing different enrichment statistics, and performing additional validation experiments. The new method provides more accurate predictions and is included in the Bioconductor package EventPointer 3.14. We tested the algorithm in four experiments involving knockdowns of seven different RBPs. The algorithm accurately assessed the statistical significance of these RBPs using only splicing alterations. Additionally, we applied the algorithm to study sixteen cancer types from The Cancer Genome Atlas (TCGA) and three from TARGET. We identified relationships between RBPs and various cancer types, including alterations in CREBBP and MBNL2 in adenocarcinomas of the lung, liver, prostate, rectum, stomach, and colon. Some of these findings are validated in the literature, while others are novel. Conclusions: The developed algorithm enhances the ability to predict and understand RBP-related splicing changes, offering more accurate predictions and novel insights into cancer-related splicing alterations. This work highlights the potential of RBPs as therapeutic targets and contributes to the broader understanding of their roles in cancer biology.

## 1. Introduction

Splicing is a crucial co- and post-transcriptional process that removes introns and splices exons to generate mature mRNA from pre-mRNA [1]. Alternative Splicing (AS) further diversifies gene expression by altering exon inclusion patterns, resulting in the production of multiple mRNA isoforms from a single gene. These isoforms can encode different proteins with distinct functions, significantly impacting cellular and organismal biology. Aberrant AS has been implicated in various hallmarks of cancer, such as angiogenesis, immortality, and immune evasion [1].

The regulation of AS is complex and orchestrated by a network of factors that includes not only the spliceosome—a dynamic assembly of small nuclear RNAs, proteins, and polypeptides—but also RNA-binding proteins (RBPs), transcription factors, epigenetic and epitranscriptomic modifications, and RNA secondary structures. Among these, RBPs play a central role by directly interacting with RNA to regulate its metabolism, methylation (such as m6A modifications), and splicing. Over 1500 RBPs have been identified in humans, many of which are integral components of the spliceosome. Given the availability of comprehensive datasets, cross-linking and immunoprecipitation sequencing (CLIP-seq) has proven invaluable for experimentally validating RBP-mRNA interactions, although it requires pre-selection of target RBPs.

RBPs are essential in cancer biology, as mutations or dysregulation in their expression can modify oncogene levels and may present potential therapeutic targets [2]. Therefore, identifying RBPs that are associated with splicing alterations is critical [3]. In our previous study [4], we created an algorithm that combines CLIP-seq data with differential splicing analysis to identify RBPs that may drive these splicing changes. This approach detects the binding of RBPs near differential splicing sites and assesses their enrichment, using only RNA-seq data for prediction. Thus, in [4] a Fisher’s test was developed to evaluate the enrichment of RBPs near differential splicing sites.

While RBPs are the focus of this study due to their pivotal role and the availability of extensive datasets, we acknowledge that additional factors such as transcriptional regulation, chromatin accessibility, and RNA secondary structure also play crucial roles in AS. A comprehensive view of splicing regulation would benefit from integrating these layers. Nonetheless, our approach represents a crucial step in understanding the RBP-specific contributions to cancer-associated splicing changes and lays the foundation for more integrative analyses in the future.

In this study, we have enhanced our previous method by incorporating the newly released POSTAR3 database [5], which includes 32% more RBP experiments for humans and mice. We have refined the algorithm for detecting differential splicing events and introduced several statistical approaches—Hypergeometric, GSEA, Wilcoxon, and Poisson Binomial—to improve the analysis of RBP enrichment. Validation using real-world data, where specific RBPs were knocked down, demonstrated consistent improvements over our earlier algorithm. Thus, the novelties of the algorithm are: (i) increased number of RBPS, (ii) improved statistics to determine which events are differentially spliced, and (iii) three new enrichment methods: Poisson Binomial, GSEA, and Wilcoxon test.

Additionally, we expanded our analysis to 19 cancer types by utilizing the TCGA and TARGET databases to identify cancer-specific RBPs. This analysis uncovered several established and novel associations, such as alterations in CREBBP and MBNL2 in lung and liver adenocarcinomas, respectively [6,7]. To promote wider scientific use, we have integrated our method into the Bioconductor platform and developed a Shiny application. These tools streamline result analysis and support the scientific community in drawing meaningful conclusions.

## 2. Materials and Methods

### 2.1. Relationship Between RBPs and Splicing Events

In this work, we started with our previously published work on Splicing Factor (SF) prediction [4]. We collected 937 CLIP-Seq experiments for 244 different RBPs contained in POSTAR3 [5]. The building of the **E × S** matrix is identical to the one described in our previous work [4]. We performed a change in genome version using the liftOver 1.30 R package [8] to transform different genome versions from human and mouse species e.g., Hg19, mm9, mm10, into a human genome hg38. With this process, we increased the sample size for the human species.

After obtaining all binding sites in hg38, we mapped the binding sites against all the splicing events from transcriptome GeneCode v.24 calculated using the EventPointer pipeline [9]. We stored this information in a sparse matrix denoted **E × S** (Events × Splicing factors). Each element denotes whether the splicing factor **j** binds to the event **i** as follows:

$$ExS_{ij}=\left\{\begin{array}{c} 1,\;\;if\;\;the\;RBP\;j\;sites\;overlaps\;the\;splicing\;event\;i\\ 0,\;\;otherwise\;\;\;\;\;\;\;\;\;\;\;\;\;\;\;\;\;\;\;\;\;\;\;\;\;\;\;\;\;\;\;\;\;\;\;\;\;\;\;\;\;\;\;\;\;\;\;\;\;\; \end{array}\right.$$

We have added some changes to improve the performance of the algorithm and integrated it into a Bioconductor R package. This version enables us to use the Fisher’s Exact Test, GSEA, a Wilcoxon test, and a new approach developed by us: the Poisson Binomial Enrichment.

### 2.2. Event Statistics

Using **E × S** it is possible to perform an enrichment analysis on the differentially spliced events. We implemented EventPointer 3.0 bootstrap statistics for alternative splicing events detector. The main strength of the pipeline is the fact that it estimates the Ψ distribution for each event using bootstrap resulting in a very robust pipeline.

### 2.3. Methodology for RBP Enrichment and Ranking

Our method outputs a ranking with the most likely enriched RBPs. This ranking is performed using four different enrichment methods. We describe these methods in the following paragraphs.

#### 2.3.1. Fisher’s Exact Test

Fisher’s Exact Test was already described in [4]. The Fisher test is based on a hypergeometric variable to calculate the probability of seeing an abnormal number of events that are differentially spliced and bound to an RBP (**Equation (1)**).

(1) PX≥k=∑X=k K KxM−Km−xMm

where,

- **M** is the total number of events.
- **K** is the number of selected events.
- **m** is the number of events regulated by RBPi.
- **k** is the number of events within K regulated by RBPi.

We include in the current work two different options to select the relevant splicing events: select the splicing events with a *p*-value under a threshold (*p*-value = 0.001) or select the first 1000 splicing events ranked by *p*-value. We used the first option in our pipeline. Additionally, users can use FDR to set the threshold (see https://github.com/JFerrer-B/SFPointer –URL accessed on 7 November 2024)

#### 2.3.2. Poisson Binomial

From **E × S** matrix we compute the probability of a specific event being regulated by a specific RBP. The event-RBP regulation probability was estimated using the methodology proposed by [10]. This methodology demonstrates that, assuming the independence between events and RBPs, the probability of an event i being regulated by an RBP j (P*ij*) can be written as p_ij=e^(μ_i+λ_j)/(1+e^(μ_i+λ_j)) (for more details see [10]). Then, this approach uses a logistic regression as depicted in **Equation (2)**:

(2) $$L\left(\mu ,\lambda \right)=\prod_{i,j}\frac{n_{ij}!}{y_{ij}\left(n_{ij}-y_{ij}\right)!}p_{ij}^{y_{ij}}{\left(1-p_{ij}\right)}^{\left(n_{ij}-y_{ij}\right)}$$

where:

- **p_ij_** is the probability of event *i* being regulated by RBP *j*.
- **p_ij_** can be written as $p_{ij}=\frac{e^{\left(\mu_{i}+\lambda_{j}\right)}}{1+e^{\left(\mu_{i}+\lambda_{j}\right)}}$ as depicted above.
- **n_ij_** is the total number of cases: by construction is equal to 1.
- **y_ij_** is equal to one if event *i* is regulated by RBP *j*, i.e., y_ij_ = {0,1}.

Then, we calculate the probability of observing an abnormal number of events that are differentially spliced and regulated by an RBP. This probability is computed with the Poisson Binomial Distribution (**Equation (3)**):

(3) $$Pr\left(X\geq k\right)=\sum_{x=k}^{K}\sum_{A\in F_{x}}\prod_{i\in A}p_{i}\prod_{j\in A^{c}}\left(1-p_{j}\right)$$

where,

- **F_x_** is the subset of *x* integers possible. if total number of elements is 3 and *x = 2* then F_2_ = {{1,2},{1,3},{2,3}}.
- **K** is the number of selected events.
- **p_i_** is the probability of an event *i* being regulated by a Splicing Factor.
- **P_j_** is the probability of an event *j* being regulated by a Splicing Factor.

This is solved using Rediscover 0.32 [10], which uses the poibin 1.6 R package to compute the *p*-values.

#### 2.3.3. Gene Set Enrichment Analysis (GSEA)

GSEA is a successful enrichment analysis method initially described in [11]. GSEA is a non-parametric test based on the Kolmogorov–Smirnov statistic that compares the distributions of a variable (usually a *p*-value, but other possibilities are also valid) between the analytes (usually genes) that have a characteristic (usually a GO annotation) and those that do not have the characteristic. The application of RBP analysis is straightforward. The variable is the *p*-value of the splicing event and the characteristic is the presence or absence of an annotated RBP binding site in the neighborhood of the event.

We have used the R-package fgsea 1.32. It allows us to “quickly and accurately calculate arbitrarily low GSEA *p*-values for a collection of gene sets” [12] by using an adaptive multi-level split Monte Carlo scheme. Despite being the fastest available, it is still slower than any of the other implemented methods. One of the advantages of GSEA is that it does not require setting a threshold on the *p*-value to state which are the significant events.

#### 2.3.4. Wilcoxon’s Test

The Wilcoxon test can also be used to perform an enrichment analysis. A Wilcoxon test is a non-parametric test that compares the medians of two data sets. In this case, given the *p*-values obtained from EventPointer, the distributions to be compared are the *p*-values of the event annotated with an RBP binding site with the *p*-values of the events not annotated with the same RBP binding site. The final result is the ranking of the RBPs with the lowest Wilxon’s test *p*-value.

The Wilcoxon test does not either require setting a threshold on the *p*-values. Our implementation (which uses sparse matrices and linear algebra) is the fastest of all the methods.

### 2.4. TCGA and TARGET Analysis

We run the SFpointer pipeline using EventPointer 3.0 and the four enrichment analyses. For the alternative splicing analysis, we selected the top five differentially spliced events for each condition and extracted the delta PSI for each event to plot the comparative analysis.

Regarding the SFpointer enrichment analysis, we extracted the RBPs that were present in at least five different cancer conditions and clustered them using Kmeans with 10-fold validation in two different groups. Additionally, we clustered the different cancer sites into three different groups using Kmeans and 10-fold cross-validation provided by the R package ComplexHeatmap 2.22 [13].

Finally, with the obtained 22 RBPs present in more than 5 cancer conditions we used the STRING MCL approach to plot and cluster the network, using STRING database information [14]. The MCL inflation parameter was set to 3.

## 3. Results

The primary achievement of this study is the development of SFpointer, a novel algorithm designed to identify RBPs that are significantly enriched in regions associated with differential splicing events. This is accomplished by integrating multiple CLIP-seq databases into a unified resource, enabling comprehensive enrichment analysis of RBPs. The input is the RNA-seq analysis of several experiments. The output is a prioritized list of RBPs that are more likely drivers of alterations in splicing patterns across the experiments, providing valuable insights for further biological validation.

The development of SFpointer involved several key challenges: (1) *Limited CLIP-seq Usage*: Given that CLIP-seq is less commonly utilized than RNA-seq, the accuracy of predicting splicing factor binding motifs is heavily reliant on the availability of CLIP experiments, necessitating extensive database integration, (2) *Statistical Significance*: The reliability of our findings is contingent upon both the quality of splicing event calls and the statistical methods employed in the enrichment analysis, (3) *Validation Requirements:* The results from our statistical pipeline must be validated against experiments with known ground truth.

We prioritized user-friendliness in the algorithm’s design to ensure accessibility for the scientific community. By successfully addressing these challenges, we have created a robust tool that enhances researchers’ ability to explore the role of RBPs in splicing regulation.

### 3.1. Included RBPs Are Increased by 30% with the Updated Databases

In our updated version, we have integrated POSTAR3, a comprehensive CLIP-seq database containing 1445 experiments across seven species, covering 348 RBPs. We included experiments mapped to the human and mouse genomes due to their genetic similarities, with mouse data converted to human genome coordinates using liftover [8]. This increased the total number of RBPs included in our analysis to 244, a 25% increase over the previous version [4].

This integrated database provides genomic loci mapped to the human genome (hg38), facilitating the identification of potential splicing events. To explore the relationship between RBP binding sites and splicing events, we constructed an indicator sparse matrix, termed **E × S** (Events × Splicing Factors), which indicates whether an RBP binding locus is within a 400 nt window of a splicing event. A detailed methodology for the construction of the **E × S** matrix can be found in our previous publication [4].

### 3.2. Boosting Sensitivity and Specificity Through a New Statistical Modeling

**Figure 1:**
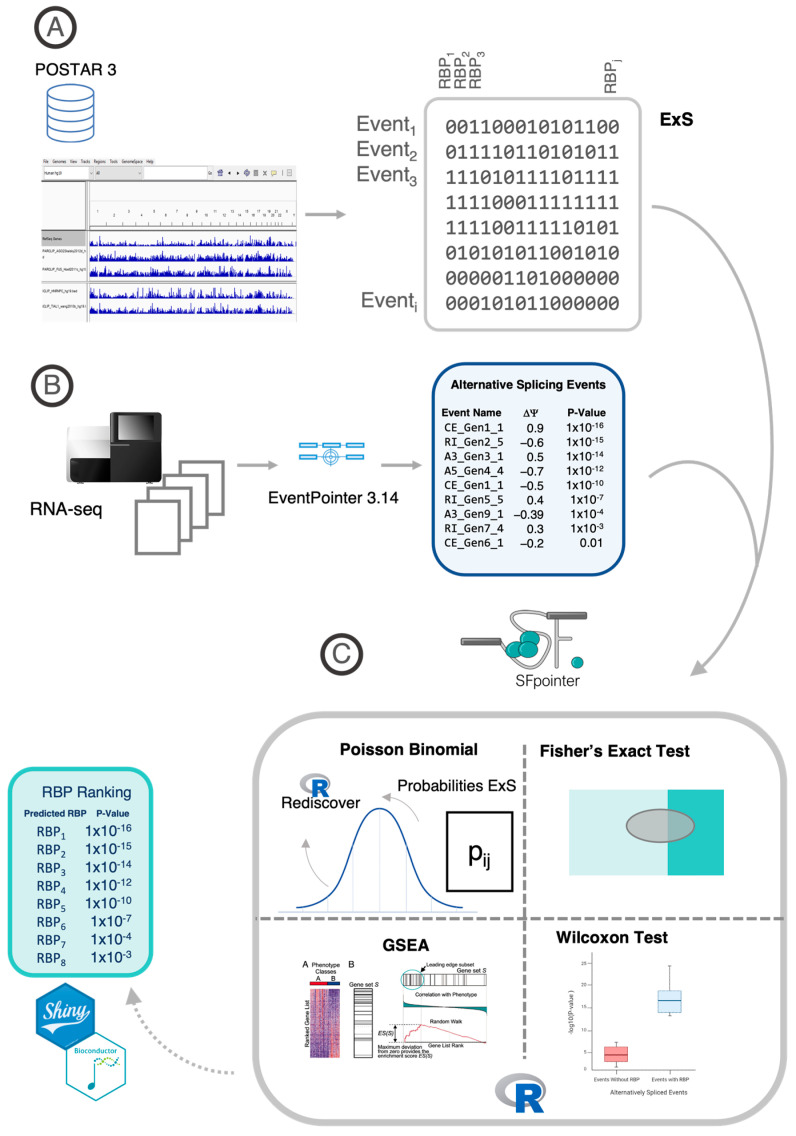
SFpointer Pipeline. (**A**) the **E × S** matrix is built from POSTAR3 CLIP experiments, where each entry i, j is 1 if RBP “j” binds near splicing event “i” annotated in the reference transcriptome and 0 otherwise. (**B**) the differentially spliced events are detected using a bootstrap version of EventPointer 3.14. (**C**) SFpointer uses these events and the **E × S** matrix to estimate RBP enrichment by applying one of four methods: Poisson Binomial, Fisher’s Exact Test, GSEA, or Wilcoxon Test, resulting in a ranked list of RBPs with enrichment *p*-values. This method is implemented as a Shiny app and in Bioconductor.

In our analysis, we observed that accurate identification of altered splicing events significantly affects the performance of splicing factor (SF) calculations. Careful selection of differential splicing events improves the accuracy of RBP enrichment. To improve this aspect, we have adopted a bootstrap-based statistical approach implemented in the EventPointer 3.14 package (Figure 1B), which increases the sensitivity compared to the previous version [9].

**Figure 2:**
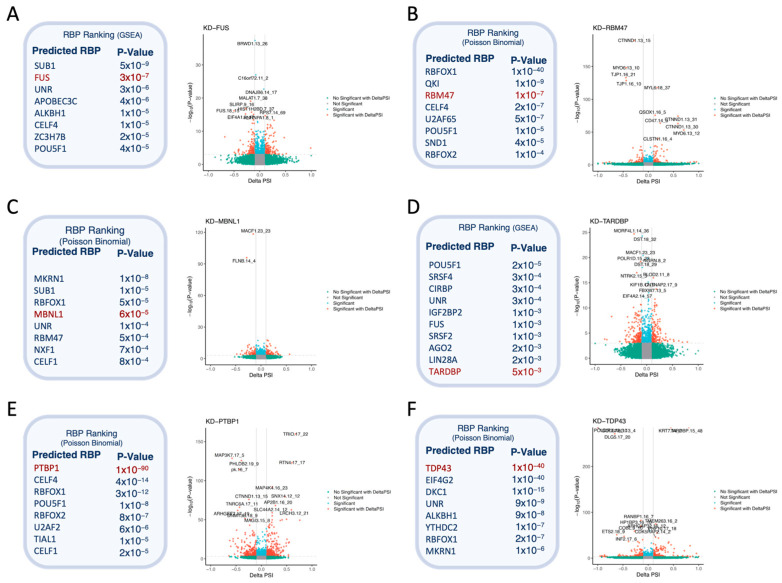
RBP ranking and volcano plot include all experiments described in Table 1 but KD-TAF15 due to its low impact on alternative splicing. (**A**) corresponds to the knock-down of FUS, (**B**) to the knock-down of RBM47, (**C**) to the knock-down of MBNL1, (**D**) to the knock-down of TARDBP, (**E**) to the knock-down of PTBP1, and (**F**) to the knock-down of TDP43. For each condition, the top 8 RBPs (top 10 in KD-TARDBP) and their enrichment *p*-values are reported, using the method that optimizes the RBP’s ranking. Each volcano plot displays in red the AS events with an absolute Delta PSI (ΔΨ) greater than 0.1 and a *p*-value lower than 1 × 10^−3^. Significant events with smaller ΔΨ changes are shown in blue, and events with large ΔΨ changes but not significant are shown in green.

In addition, we have implemented four different statistical enrichment analyses on the AS events to predict the differential activity of the RBPs (Figure 2). The four methods are: Fisher’s exact test, Poisson Binomial, GSEA, and Wilcoxon test. As mentioned before, these algorithms assess which RBP binding motifs are overrepresented in regions with altered splicing events.

The first method (Fisher’s exact test) is based on the hypergeometric distribution. This test estimates, using the **E × S** matrix, the enrichment of the RBPs by setting a threshold on the *p*-values to state which are the differentially spliced events. This method is consistently used to perform GO enrichment analysis and was already implemented in the previous version of the algorithm [4,15,16].

Note that, the data used for enrichment analysis (the **E × S** matrix) is a potential source of bias because some RBPs bind to a large proportion of splicing events while others bind to very few. Furthermore, certain splicing events may have numerous RBP hits while others have minimal hits. A similar statistical analysis was performed in Discover [17] for the detection of mutually exclusive mutations and showed that variations in the density of rows and columns in the input matrix (in our case **E × S**) can introduce bias in naïve analyses based on the hypergeometric distribution.

To address this bias, we developed the Poisson Binomial method. For this, we used Rediscover [10], an R package that implements the Poisson Binomial distribution instead of the standard hypergeometric distribution.

Importantly, both the hypergeometric and Poisson Binomial methods require the user to select a threshold to determine when a *p*-value is considered significant. I.e., neither of these methods fully exploits the ranking of aberrant AS events; for example, a splicing event ranked first is treated the same as one ranked last, provided its *p*-value is below the threshold. To improve this analysis, we incorporated Gene Set Enrichment Analysis (GSEA) [11], which is based on the Kolmogorov–Smirnov test and effectively exploits ranking information, demonstrating strong statistical power in GO enrichment analysis. Finally, we also included a standard Wilcoxon test, a non-parametric method that similarly exploits the ranking of events.

### 3.3. SFpointer Accurately Identifies the RBP Causing Splicing Disruption

We evaluated the proposed pipeline using four RNA-seq experiments with seven different knocked-down RBPs, which allowed us to assess its accuracy (Figure S1). To facilitate a fair comparison, we reran these experiments using the previous version of SFpointer, using the current **E × S** matrix from our updated algorithm (Table 1). The primary goal of this comparison was to determine whether the improvements in precision and sensitivity were due to changes in the selection of affected splicing events, updates to the enrichment statistics incorporated into the algorithm, and the expanded data set available from POSTAR3. Detailed results of the enrichment analyses are presented in Supplementary Tables S3–S8. In all cases, we considered events with a *p*-value less than 0.001 to be significant (Table S2).

We revisited the analysis presented in reference [4], which evaluated the ability of the previous algorithm to identify RBPs from the GSE77702 dataset. In this dataset, we compared three different contrasts: KD-FUS, KD-TARDBP, and KD-TAF15 against scramble transfection [18]. For the KD-FUS contrast, we observed a significant improvement, with its ranking advancing from 11th to the top position, highlighting the improvement of POSTAR3 over POSTAR2. In the case of the KD-TARDBP condition, expression analysis indicated that the knockdown of TARDBP was incomplete, resulting in an insufficient reduction in gene expression levels (Figure S2). Conversely, the KD-TAF15 condition was excluded from the original study because of the minimal effect of TAF15 on alternative splicing regulation as previously demonstrated [4,5]. In the original results using the POSTAR2 database, TARDBP ranked 20th, while TAF15 was not identified as a significant RBP. After re-analysis using POSTAR3 as the reference database, TARDBP was ranked 68th and TAF15 was ranked 125th. Although the analysis with POSTAR3 changed their rankings, both remained as non-significant, consistent with the previously mentioned limitations.

We also analyzed three additional datasets: (i) PRJEB39343, in which three RBPs (PTBP1, ESRP2, and MBNL1) were knocked down in gastric cancer cell lines [19], (ii) GSE136366, in which TDP43 was knocked down in HeLA cell lines [20], and (iii) GSE75491, in which RBM47 was knocked down in H358 cell lines [21]. For the PRJEB39343 dataset, we excluded the KD-ESRP2 condition because ESRP2 is not included in the current **E × S**.

We compared the new version of SFPointer using Fisher’s exact test (Table 1, third column) with the previous version, also using Fisher’s exact test (Table 1, second column). Notably, the ranking of MBNL1 improved from 81st to 9th. For TAF15 and TARDBP, no significant improvement was observed, which is to be expected given the conditions previously discussed. For the other knockdowns, there was little variability and their rankings remained high. These differences can be attributed to the new SFPointer using the latest version of EP, which is more accurate in identifying differentially spliced events between conditions.

In addition to the traditional Fisher method for enrichment analysis, we introduced three new approaches–GSEA, Wilcoxon, and Poisson Binomial—that improve accuracy in all scenarios. In experiments where the targeted RBPs (PTBP1, MBNL1, FUS, TDP43, and RBM47) were effectively knocked down, these RBPs were consistently in the top 10% across all methods, demonstrating the robust detection capability of SFPointer. In particular, the Poisson binomial method yielded excellent results, accurately ranking the knocked-down RBPs within the top 4 out of 244 positions (Table 1). Full results of the enrichment analyses are provided in Supplementary Tables S3–S8.

As expected, the results are highly dependent on the quality of the experiments. The alteration in alternative splicing is small, RBP does not achieve a high ranking, as seen with KD-TAF15 and KD-TARDBP. Interestingly, TARDBP and TDP43 refer to the same gene, but their ranking results differ significantly between GSE77702 and GSE136366, highlighting the influence of experimental quality on enrichment results. In GSE77702, TARDBP expression decreases almost twofold, whereas in GSE136366 it decreases tenfold (Figures S3 and S7).

Finally, for each KD experiment, Figure 2 includes an AS analysis result that illustrates the specific AS changes for each condition that are likely related to the splicing regulatory activity of the RBP. For example, BRWD1 and MALAT-1, which show significant decreases in Ψ in the KD-FUS condition (Figure 2A), have previously been implicated in FUS activity [22]. In addition, FLNB in KD-MBNL1 (Figure 2C) has been described as part of the MBNL1-mediated apoptosis pathway [23], and MAPK kinase genes in KD-PTBP1 (Figure 2E) have been reported as inhibitors of the MAPK/ERK pathway [24].

### 3.4. Analysis of the ENCODE Database

The application of SFPointer to the ENCODE dataset involved the analysis of 212 experiments related to the knockdown of 106 different RBPs in HEGP2 and K562 cell lines. Most of these experiments included only two control and two knockdown samples, with shared control samples utilized across multiple experiments. Specifically, the HEGP2 experiments employed 21 distinct types of control sample sets, while the K562 experiments used 29 types.

However, the results obtained from SFPointer were suboptimal. This is likely attributed to the presence of other differentially expressed RBPs in addition to the targeted knockdown RBP (Figure S9). Furthermore, when examining the ΔΨ values, it became evident that the experiments clustered more significantly based on the control sample sets rather than the knockdown effects (Figures S10–S13). This suggests that factors such as the choice of control samples may account for the observed differences in splicing, rather than the intended knockdown of the specific RBP.

This analysis highlights the importance of considering experimental design and control sample selection when interpreting results from RBP knockdown studies, as they can significantly influence the outcomes and conclusions drawn from the data

### 3.5. Pan-Cancer Analysis of Splicing Regulators Reveals Three Groups of Tumors with Similar RBPs Profiles

**Figure 3:**
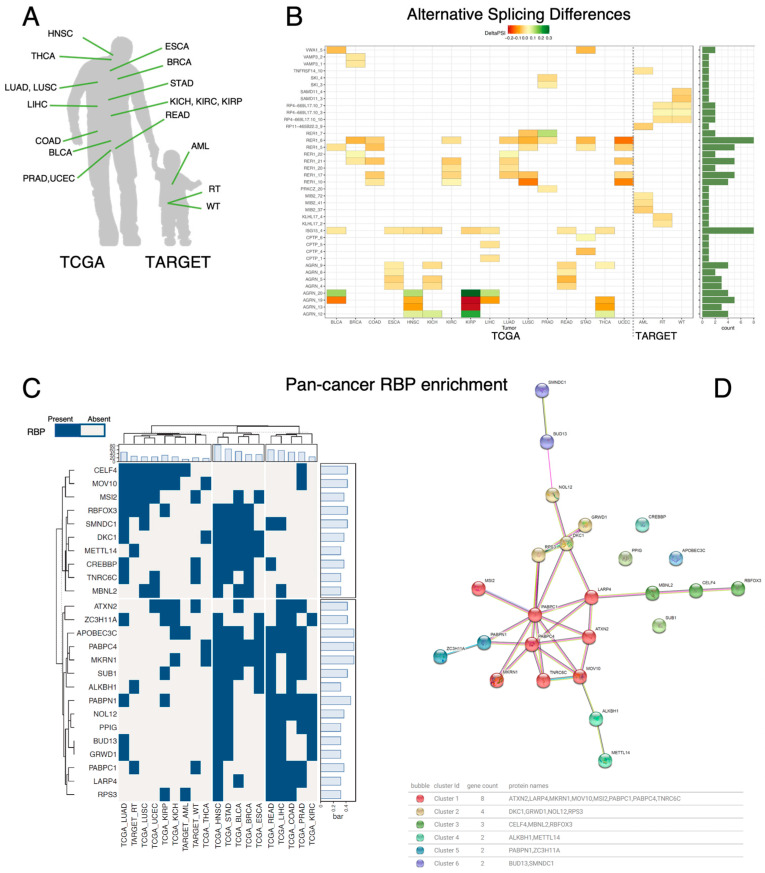
(**A**) Tumor types from TCGA and TARGET included in the study, focusing on those with sufficient normal samples. (**B**) Heatmap of pan-cancer alternative splicing analysis, showing the top five significant splice events per cancer type. The x-axis lists cancer types, the y-axis lists splicing events, with red indicating negative ΔΨ and green positive ΔΨ. (**C**) Heatmap of 22 RBPs enriched in over 5 tumor types, clustered into two groups, with enrichment shown in blue; includes bar charts of RBP abundance and RBP count per tumor type. (**D**) STRING clustering of RBPs, with colors indicating clusters, bubbles representing RBPs, and lines showing STRING relationship evidence; includes a cluster description table.

Several studies have demonstrated the significant role of aberrant splicing in cancer development [3,15,25]. Using SFPointer, we conducted a comprehensive pan-cancer AS study to investigate the role of RBPs in driving aberrant aAS across 19 different cancer types, utilizing data from 9514 patients sourced from the TCGA and TARGET databases (Figure 3A). The results presented in Figure 3 were obtained using only the Poisson Binomial approach. Finally, we clustered the most frequently identified RBPs using STRING [14]. For an analysis of the biological impact of splicing events, see [9].

Figure 3B shows the top five AS events for each cancer type from the TCGA and TARGET datasets. Notably, several AS events recur across cancer types, while AS events in childhood cancers are highly specific to each type, with no shared AS events between these and adult cancers. In contrast, two genes—AGRN (ENSG00000188157) and RER1 (ENSG00000157916)—are recurrently differentially spliced in adult tumors, consistently appearing in the top five positions.

AS events involving AGRN are present in four out of sixteen adult cancers: ESCA, KICH, READ, and THCA. AGRN is a gene known for its tissue-specific isoform expression and has recently been implicated in the Hippo pathway in the tumor microenvironment in several cancer types [26,27]. Its aberrant splicing is associated with impaired neuromuscular junction synaptogenesis [28], although no current studies directly link its splicing to tumorigenesis.

In the case of RER1, AS events in this gene ranked in the top 5 in 9 out of 16 tumor types. Interestingly, the RER1 gene has been associated with colon and pancreatic cancer [29,30]. Specifically, one of its AS events has been reported to be associated with disease recurrence in colorectal cancer [30], and its biological function has been reported to induce carcinogenesis in pancreatic cancer [29].

Furthermore, using the results obtained by SFpointer for each cancer site, we selected the RBPs that appeared to be significantly enriched in at least five different cancer types. We performed k-means clustering by RBPs and cancer types with 10-fold cross-validation. The results are shown in Figure 3C. We clustered these results by both columns (cancer types) and rows (enriched RBPs). The column clustering shows three different clusters of cancer types according to the number of RBPs disregulated in each condition. The top bar graph shows the number of enriched RBPs for each cancer type. The middle cluster shows that HNSC, STAD, BLCA, BRCA, and ESCA are the tumor types with the highest number of altered RBPs. They all have in common the enrichment of splicing sites regulated by DKC1, METTL14, PABPC4, and MKRN1. These RBPs have a strong relationship with cancer development, e.g., DKC1 is related to the expression of tumor suppressors [31], METTL14 mediates tumor progression through SOX4 alteration and WTAP [32], PABPC4 is downregulated in metastatic cells [33], and MKRN1 modulates tumor progression through the AKT pathway [34].

The second cluster (rightmost group) includes relevant cancer types such as COAD or READ and shares the enrichment of PABPN1 and NOL12, both of which are related to tumor progression [35,36]. Finally, the third group (leftmost group) includes the tumors with the lowest number of dysregulated genes. This group is characterized by the high presence of altered CELF4 and MOV10 among its samples. Both genes have been implicated in carcinogenesis [37,38].

Regarding the clustering by rows (RBPs), there are two main clusters: the first one (mostly related to AS alterations in adult cancer), the bottom cluster in the plot, includes relevant cancer genes such as MKRN1, DKC1, or PABPC4. The second cluster seems to modulate AS at more tissue-specific sites (top part of the plot) and includes relevant oncogenes such as CREBBP [6] or MBNL2 [7].

Finally, these 22 RBPs were clustered the RBPs using STRING MCL methodology [25], finding 6 clusters shown in Figure 3D and Supplementary Table S9, e.g., cluster 1 includes LARP4, ATXN2, MKRN1, PABPC1, PABPC4, MOV10, TNRC6C, and MSI2 RBPs; and cluster 2 contains DKC1, NOL12, GRWD1, and RPS3 RBPs. We observed that 13 out of 19 (about 70%) of the RBPs included in the largest STRING clusters were included in the same group by our method, suggesting that our approach can find functional relationships among RBPs.

### 3.6. Constructing a Pan-Cancer Splicing Regulator Resource

To facilitate the exploration of our findings, we developed a Shiny application that integrates the results of our pan-cancer RBP enrichment and AS analysis. This application is accessible at https://biotecnun.unav.es/app/SFPointer (accessed on 7 November 2024) and allows users to select specific cancer sites while providing a comprehensive ranking of 244 RBPs across 19 different tumor types derived from the TCGA and TARGET databases.

The Shiny app enables users to view the results of alternative splicing analysis for each of the 16 conditions shown in Figure 2A, along with the ability to visualize specific splicing events. In addition, it includes enrichment results for each RBP, allowing users to query the data both at the RBP level—to see which tumors exhibit enrichment—and by condition, to see all RBPs enriched in a particular tumor type. Users can also download graphs and tables directly from the app and perform survival analyses based on each RBP in relation to tumor types, providing insight into the relevance of each RBP in contributing to overall survival.

In addition, the underlying code of SFPointer has been integrated into the EventPointer package already available in the Bioconductor repository. For those interested in the technical details, the code vignettes and a model of the pipeline can be found at https://github.com/JFerrer-B/SFPointer (accessed on 7 November 2024). This integration not only improves accessibility but also supports researchers in further exploring the implications of our findings in the context of alternative splicing and cancer biology.

## 4. Discussion

In this study, we have developed and implemented a new method to detect potential RBP drivers at AS in different biological conditions. Results can be directly inferred from an RNA-seq experiment allowing us to calculate the disruption of 244 RBPs—avoiding the need for performing 244 CLIP experiments. Furthermore, SFpointer has been validated using seven different KD experiments. The results of the validation presented the disrupted RBP in the top five of predicted ones and outperformed the previous methodology. Finally, we have applied it to TCGA and TARGET discovering pan-cancer actuation RBPs and new cancer-specific RBPs that have been made available for consultation by any user through our SFPointer 1.0 Shiny app.

Our software is a statistical method that is based on co-occurrence, but we are aware that it does not imply causality. We cannot claim, using the plain results, that the predicted RBPs are causing the observed splicing changes. It is a method that only states that the genomic loci where some particular RBPs bind, are especially enriched in places where there is differential splicing. Henceforth, it provides an educated guess to perform some type of biological validation of the involved RBPs.

In reference to the above, the method relies for its predictions on the **E × S** matrix that relates the genomic loci of the RBPs with the alternative splicing events of the transcriptome. This matrix was constructed using all the human and mouse experiments from POSTAR3. It includes CLIP experiments from many different conditions and tissues were stored in the database. However, apart from translation to GRCh.38, no further normalization was performed. Thus, SFpointer predicts over a particular tissue experiment using information from cell lines of other tissues, which is debatable since each tissue has a very different behavior. However, we have prioritized predictive ability over prediction accuracy, i.e., for a certain condition we prefer to have the possibility to give a result than to reduce the predictive ability to one or two RBPs, because of the scarcity of CLIP experiments performed on those cell lines. In six out of the seven experiments, this approach proved to be valid.

Finally, regarding enrichment methods, we have included in our tool most of the state-of-the-art methods: Fisher’s Exact Test, Poisson Binomial, GSEA, and Wilcoxon. The first two methods do not consider the rankings of events with alternative splicing, while the latter does. As expected, the results of the four methods are quite similar, and all of them perform reasonably well. We noticed that the precision of the RBP prediction strongly depends on the conditions of the experiment—i.e., *TARDBP* is predicted in 10th and 1st position in two different KD-*TARDBP* experiments being the first a less effective KD of *TARDBP*—and in these conditions, the enrichment methods tend to differ in the results obtained. The robustness of the AS analysis will also considerably affect the result, we recommend the use of EventPointer as its results are robust and we have been able to validate them, e.g., by identifying the MEK pathway with *PTBP11*.

Regarding the validations using seven KD experiments, we applied each of the four methods and the results demonstrate that the statistical advances presented in this work improve the results obtained with the previous version of SFpointer. Indeed, we observe that in the experiments with almost perfect KO of the RBP, the enrichment results place the RBP in the top five of the ranking of alerted RBPs. Remarkably, the four enrichment methods provide similar predictions in each condition. Although GSEA enrichment and Poisson Binomial especially stand out for their performances, the former is one order of magnitude slower, but both are equally accurate and have obtained the best qualitative result of the validations.

Despite our efforts to apply SFPointer on the ENCODE dataset, we were not able to get proper results. There can be several reasons for this. First of all, most of the experiments only include two control and two knockdown samples, and these control samples are shared across multiple experiments. We observed that the experiments clustered together based on the control samples (Supplementary Figures S9 and S11). The correlation between experiments with the same control is stronger than those with different controls (Supplementary Figures S10 and S12). This result is completely unexpected since using the ΔΨ should cancel out the effect of having the same reference, as the ΔΨ is a relative value.

In addition, the knockout seems to be unspecific: in all instances of the experiment with the HEPG2 cell line, more than one RBP show exhibited differential expression (*p*-value < 0.001). A similar result appears with the K562 cell line with 94/106 experiments showing differential expression for more than one RBP. We even found that the most under-expressed RBP was not the knocked out RBP in 56/106 and 38/106 cases for HEPG2 and the K562 cell lines, respectively. As a result, we have not included these results in the main manuscript, but in the Supplementary Materials.

A major contribution of this article is the application of the SFpointer pipeline to all data from both TCGA and TARGET. The results obtained are very promising: we have achieved the identification of two co-occurring splicing events present across different tumor types. This fact highlights the relevance of the study of splicing concerning cancer, and how it could be possible to include splicing events as biomarkers [39,40]. Isoform-specific data was downloaded from [41], which used GENCODE24 as the reference. This is the reason why we used a somewhat older version of the transcriptome.

Likewise, we have performed an enrichment in RBPs for the different tumor types, obtaining three different groups of behavior depending on the number of RBPs in which they are enriched, having a special variability of splicing in tumors such as BRCA, HNSC, while pediatric tumors or lung cancer have much less variability in the enrichment of RBPs. The presence of *PABPC4* and *MKRN1* has been observed as the most frequently enriched RBPs in the different types of cancer coherently with the literature [33,34], proving the relevance of this approach.

In addition, approximately 70% of the RBPs predicted with our methodology cluster similarly using STRING data and analytics. Interestingly, using completely different information, we have deduced a qualitatively similar behavior. This gives a glimpse of the statistical power of the method.

Finally, we have developed a shiny application through which it is possible to consult the results of the pan-cancer analysis, the events with which a binding site of an RBP coincides, and the RBPs that have a binding site in a given AS event. This app is available at https://gitlab.com/Jferrerb/sfpointer_gui (accessed on 7 November 2024). We have also added code and the corresponding vignettes with their explanation to Bioconductor, where it is integrated within EventPointer for use by all those researchers who wish to give a first biological interpretation of the results of their alternative splicing analysis.

While our study provides valuable insights into the role of RBPs in alternative splicing, we acknowledge a key limitation: the static nature of our computational approach may not fully capture the context-specific variability of RBP interactions. RBP effects on splicing are known to vary widely depending on tissue type, cellular state, and specific cancer context. Consequently, findings derived from general datasets may lack the specificity needed to fully represent these dynamic roles. We emphasize the importance of integrating tissue- and condition-specific RBP data in future studies to enhance the applicability of our findings across diverse cancer types. Expanding this approach to include context-specific datasets would allow for a more refined analysis, better reflecting the unique regulatory roles of RBPs in different biological and pathological environments.

## 5. Conclusions

We have improved the algorithm presented in [4] with the following novelties: (i) increased number of RBPS, (ii) improved statistics to determine which events are differentially spliced and (iii) three new enrichment methods: Poisson Binomial, GSEA, and Wilcoxon test. We observe that the improvements to the algorithm improve the accuracy compared to [4]. Among the four enrichment methods, Poisson Binomial and GSEA stand out in terms of performance.

We applied this method to 19 cancer types from TCGA and TARGET. To make these results more accessible to the scientific community, we have developed a shiny app.

Finally, this tool is easy to use for anyone who wants to analyze which RBPs are possible candidates for regulating splicing between different conditions. The algorithm is available on GitHub at https://github.com/JFerrer-B/SFPointer (accessed on 7 November 2024).

## 6. Future Lines

Our algorithm represents a foundational step toward identifying RBPs associated with AS, but we envision several directions to enhance and expand its capabilities. Currently, our approach relies on existing CLIP-seq datasets, which are constrained by the availability of RBP binding data across various tissues and conditions. As additional CLIP data becomes available, we plan to incorporate these expanded datasets to improve the specificity and applicability of our method, allowing us to capture more accurately the context-dependent roles of RBPs in splicing regulation.

In the future, we aim to develop tissue-specific **E × S** matrices to facilitate a more targeted analysis of RBP interactions within specific biological environments. This tissue-centered approach will help address some limitations of general datasets, providing insights into RBP behavior and splicing regulation unique to particular tissue contexts. Such specificity is essential for advancing our understanding of how RBPs dynamically contribute to splicing alterations in a tissue-dependent and disease-specific manner.

Additionally, EventPointer, our tool for splicing event detection, is currently undergoing improvements to enable the identification of de novo splicing events. By incorporating de novo events alongside established splicing alterations, EventPointer will allow for a more comprehensive study of RBP interactions in a condition- and tissue-specific context. This advancement could reveal novel splicing mechanisms and enhance our ability to study the intricate regulation of RBPs in specific pathological states.

Through these future developments, we aim to refine our tools to provide a more precise and context-sensitive analysis of RBPs, ultimately deepening our understanding of their roles in the complex landscape of alternative splicing and cancer.

## Figures and Tables

**Table 1:** This table contrasts the ranking positions obtained using the original version of SFpointer with the current **E × S** matrix against those generated with the four new enrichment methods using the updated EventPointer 3.14 pipeline and **E × S**. It also considers the database used, comparing POSTAR3 and its predecessor POSTAR2. The lowest ranking positions for each condition are highlighted in bold. NS stands for “not significant”. The numbers shown in red indicate the percentile ranking.

*RBP*		POSTAR2	POSTAR3				
		*SFPointer Original* *(Fisher’s Exact Test)*	*SFpointer Original* *(Fisher’s Exact Test)*	*SFpointer New (Fisher’s Exact Test)*	*SFpointer New (Poisson Binomial)*	*SFpointer New (GSEA)*	*SFpointer New (Wilcoxon Test)*
*PRJEB39343*	PTBP1	**-**	**1/**244|0.99	**1|** 0.99	**1|** 0.99	**1|** 0.99	2|0.99
*PRJEB39343*	MBNL1	-	81**/**244|0.67	9**|**0.96	**4|** 0.98	21|0.91	22|0.91
*GSE77702*	FUS	**11/195|** 0.94	**1/**244|0.99	2**|**0.99	4**|**0.98	2|0.99	2|0.99
*GSE77702*	TAF15	NS	125**/**244|0.49	**117|** 0.52	152**|**0.37	164|0.32	177|0.27
*GSE77702*	TARDBP	20/195**|**0.90	68**/**244|0.72	45**|**0.81	43**|**0.82	**10|** 0.96	30|0.88
*GSE136366*	TDP43	**-**	**1/**244|0.99	**1|** 0.99	**1|** 0.99	**1|** 0.99	**1|** 0.99
*GSE75491*	RBM47	**-**	**7/**244|0.97	8**|**0.97	**3|** 0.99	10**|**0.96	4**|**0.98

## Data Availability

RBPs binding information was extracted from a recently upgraded resource POSTAR3 [5], publicly available at -http://postar.ncrnalab.org-(accessed on 7 November 2024). Fastq files corresponding to the GSE136366 and the GSE75491 experiments were downloaded from ENA. Transcript expression was obtained from the Fastq files using Kallisto. Alternative splicing analysis was performed using the EventPointer pipeline. TCGA data were downloaded from [41], where transcript expression was computed using Kallisto and GENCODE24 as reference transcriptome. The Code of all the analyses is available in -https://github.com/JFerrer-B/SFPointer- (accessed on 7 November 2024). Results of regarding the TCGA data can be consulted in the previously mentioned shiny app -https://gitlab.com/Jferrerb/sfpointer_gui-(accessed on 7 November 2024).

## Supplementary Materials

The following supporting information can be downloaded at: https://www.mdpi.com/article/10.3390/biomedicines12112592/s1, Supplementary materials and tables are in external files. Supplementary Material file contains Supplementary Figures S1–S13 and more details of the ENCODE analysis. Suplementary tables contains Supplementary Tables S1–S9. Table S1. Table showing the ranking positions for the 7 KD-RBP conditions. The results compare the positions in the ranking using the current ExS with only the canonical alterntive splicing events. The minimum positions in ranking for each condition are highlighted in bold. Table S2. Table showing for the 7 KD-RBP conditions the number of events considered significant and the corresponding FDR. Table S3. Detailed results of the GSEA enrichment analyses corresponding to the knock-down of FUS. Table S4. Detailed results of the GSEA enrichment analyses corresponding to the knock-down of TARDBP. Table S5. Detailed results of the Poisson-Binomial enrichment analyses corresponding to the knock-down of PTBP1. Table S6. Detailed results of the Poisson-Binomial enrichment analyses corresponding to the knock-down of MBNL1. Table S7. Detailed results of the Poisson-Binomial enrichment analyses corresponding to the knock-down of TDP43. Table S8. Detailed results of the Poisson-Binomial enrichment analyses corresponding to the knock-down of RBM47. Table S9. Detailed information from the 6 clusters obtained by clustering the 22 RBPS in Figure 3. This clustering was done using the STRING MCL methodology. Figure S1. SFpointer validation across 7 independent KD experiments. Positions of the RBPs for each of their KD using the original version and four methods included in SFpointer. Fisher, GSEA, Poisson Binomial, and Willcoxon are shown in pink, purple, black, and orange respectively, and the original version of SFpointer with the previous pipeline of EventPointer but using the current *ExS* is shown in red. Each point represents the ranking position of each RBP for the different statistical approaches. The KD-*TAF15* experiment is included as evidence of the absence of alternative spicing activity. Figure S2. experiment GSE77702. The second and third samples correspond to the samples in which FUS was knocked down. Figure S3. Expression of the TARDBP gene throughout the samples of the experiment GSE77702. The seventh and eighth samples correspond to the samples in which TARDBP was knocked down. Figure S4. Expression of the TAF15 gene throughout the samples of the experiment GSE77702. The Fifth and sixth samples correspond to the samples in which TAF15 was knocked down. Figure S5. Expression of the MBNL1 gene throughout the samples of the experiment PRJEB39343. The first three samples correspond to the samples in which MBNL1 was knocked down. Figure S6. Expression of the PTBP1 gene throughout the samples of the experiment PRJEB39343. The tenth, the eleventh, and the twelfth samples correspond to the samples in which PTBP1 was knocked down. Figure S7. Expression of the TARDBP gene throughout the samples of the experiment GSE136366. The last three samples correspond to the samples in which TARDBP was knocked down. Figure S8. Expression of the RBM47 gene throughout the samples of the experiment GSE75491. The last three samples correspond to the samples in which RBM47was knocked down. Figure S9. A) Number of RBPs differentially expressed in each knockdown experiment for HEPG2 and K562 cell lines. B) Correlation between the number of RBPs differentially expressed and the number of statistically significant splicing events. Figure S10. A heatmap illustrating the correlation of ΔΨ across HEPG2 experiments. The colors indicated along the top and left borders of the graph represent the respective sets of control samples for each experiment. It can be observed that the experiments tend to cluster according to their control sample sets. Figure S11. Pearson correlation of the ΔΨ of the samples with the same control samples in blue and with different control samples in yellow. Figure S12. A heatmap illustrating the correlation of ΔΨ across K562 experiments. The colors indicated along the top and left borders of the graph represent the respective sets of control samples for each experiment. It can be observed that the experiments tend to cluster according to their control sample sets. Figure S13. Pearson correlation of the ΔΨ of the samples with the same control samples in blue and with different control samples in yellow.
