# Supplementary material for: A Systematic Identification of RNA-Binding Proteins (RBPs) Driving Aberrant Splicing in Cancer
Source: Biomedicines. 2024 Nov 13;12(11):2592. doi: 10.3390/biomedicines12112592 (PMC11591948; doi:10.3390/biomedicines12112592)
Supplement: Supplementary file 1 [file biomedicines-12-02592-s001.zip › Supplementary_Material.pdf]

Supplementary Material: A Systematic Identification of RBPs Driving Aberrant Splicing in Cancer

Cesar Lobato-Fernandez †, Marian Gimeno †, Ane San Martín, Ana Anorbe, Angel Rubio * and Juan A. Ferrer-Bonsoms *

Departamento de Ingeniería Biomédica y Ciencias, TECNUN, Universidad de Navarra, 20009 San Sebastián, Spain; clobatofern@unav.es (C.L.-F.); mgimenoc@unav.es (M.G.); asan.27@alumni.unav.es (A.S.M.); aanorbe@alumni.unav.es (A.A.)

* Correspondence: arubio@unav.es (A.R.); jafhernandez@unav.es (J.A.F.-B.)

† These authors contributed equally to this work.

SUPPLEMENTARY MATERIAL

1 SFPointer accuracy

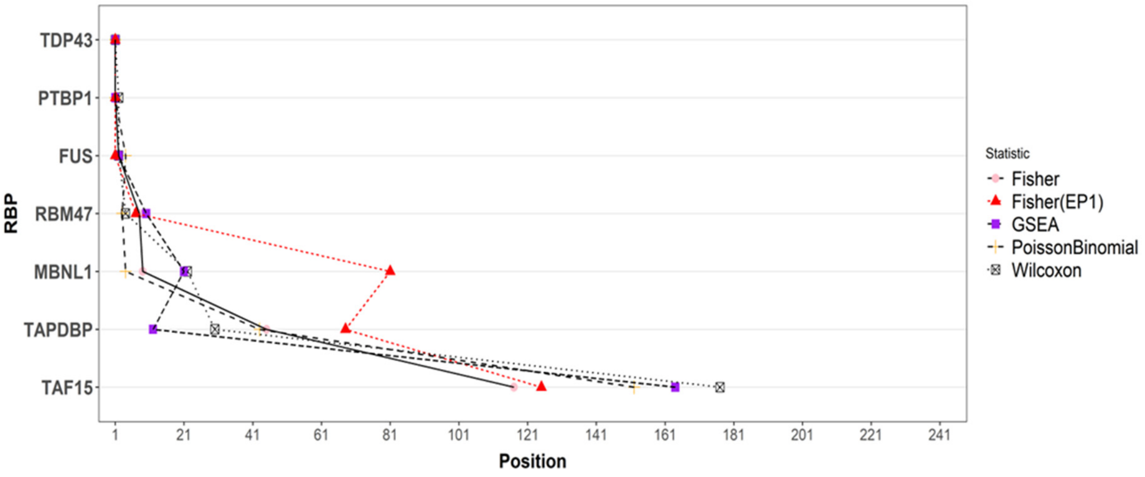

Figure S1. SFpointer validation across 7 independent KD experiments. Positions of the RBPs for each of their KD using the original version and four methods included in SFpointer. Fisher, GSEA, Poisson Binomial, and Willcoxon are shown in pink, purple, black, and orange respectively, and the original version of SFpointer with the previous pipeline of EventPointer but using the current *ExS* is shown in red. Each point represents the ranking position of each RBP for the different statistical approaches. The KD-*TAF15* experiment is included as evidence of the absence of alternative spicing activity.

2 Supplementary Figures of KD expression

2.1 GSE77702

The following pictures show the expression of FUS, TARDBP, and TAF15:

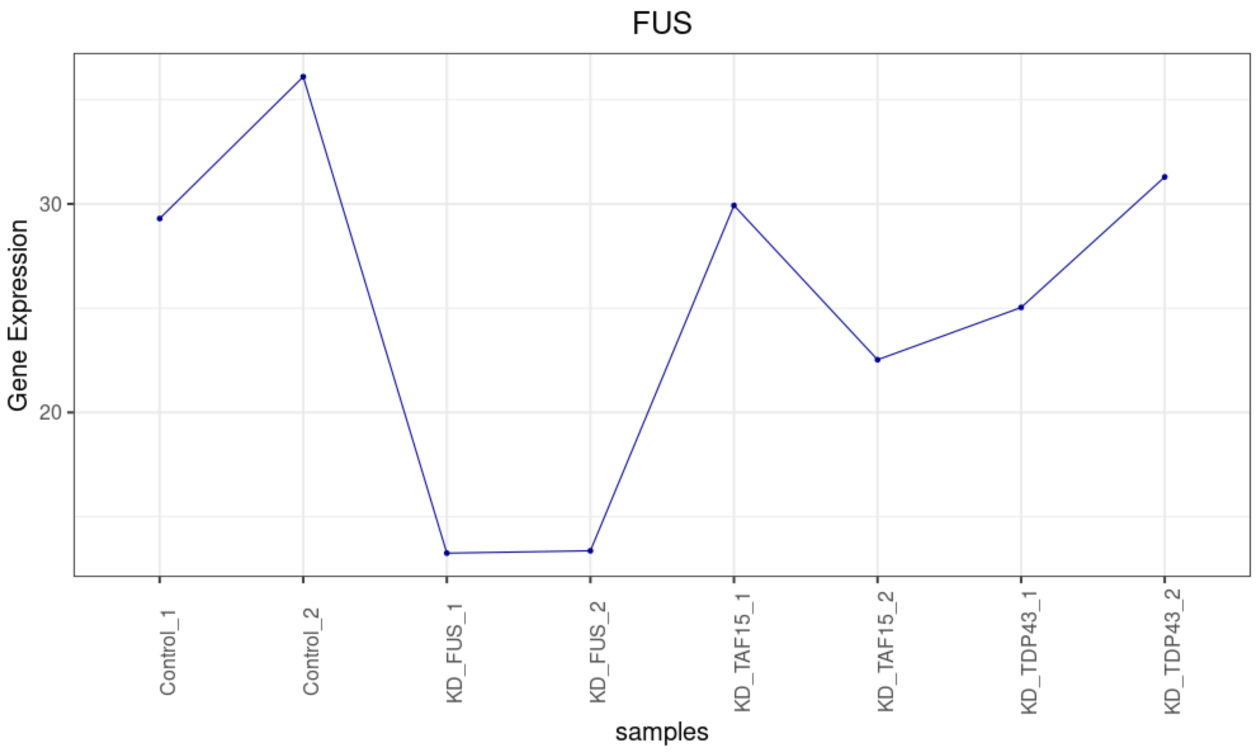

Figure S2. experiment GSE77702. The second and third samples correspond to the samples in which FUS was knocked down.

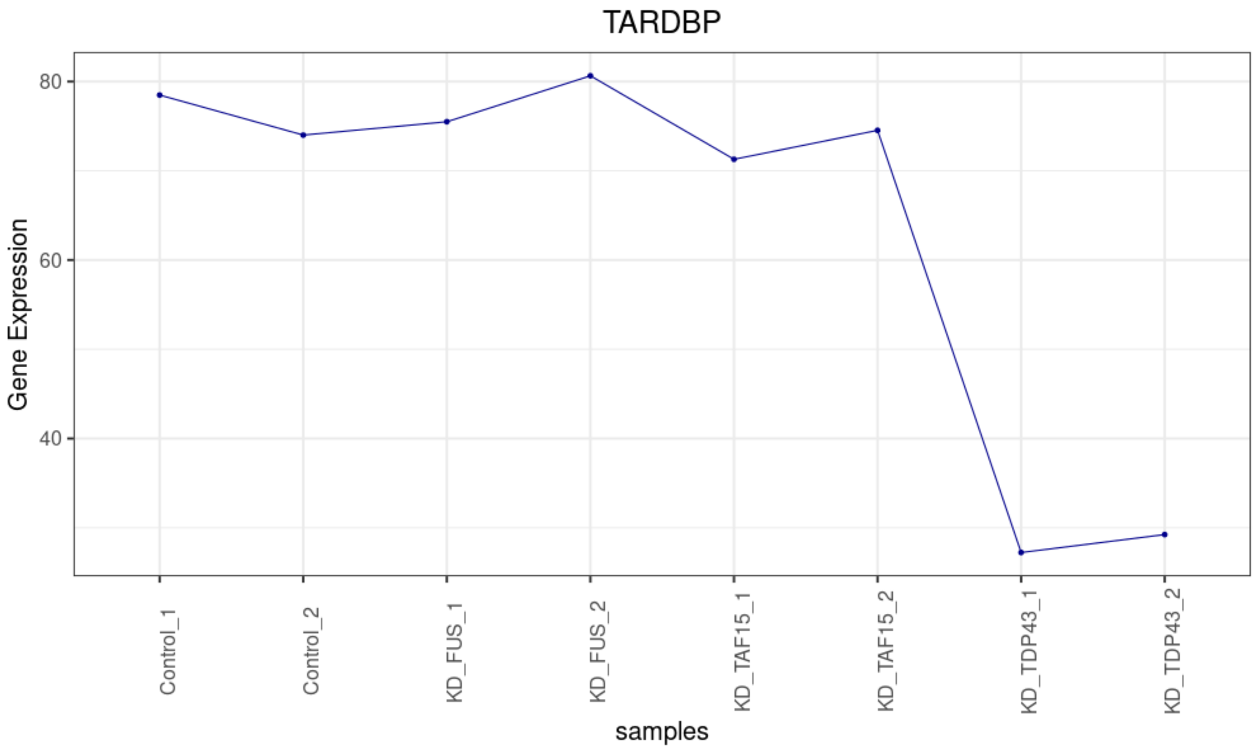

Figure S3. Expression of the TARDBP gene throughout the samples of the experiment GSE77702. The seventh and eighth samples correspond to the samples in which TARDBP was knocked down.

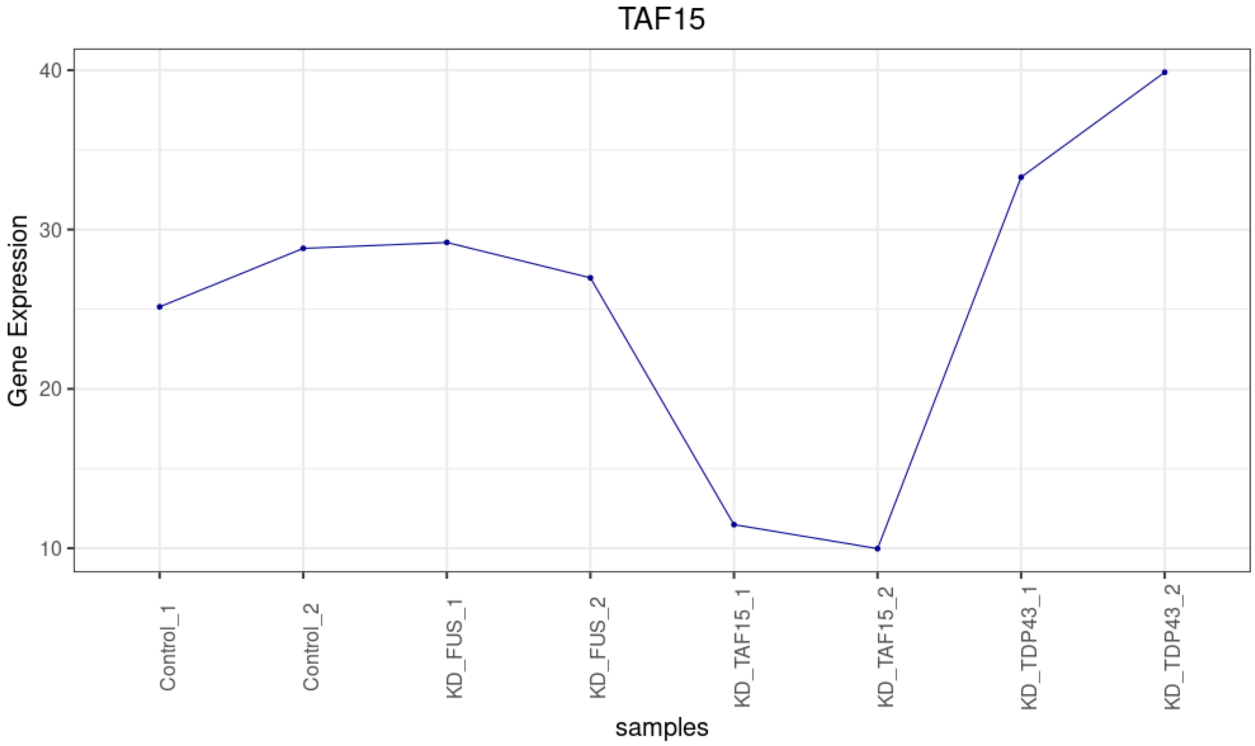

Figure S4. Expression of the TAF15 gene throughout the samples of the experiment GSE77702. The Fifth and sixth samples correspond to the samples in which TAF15 was knocked down.

PRJEB39343

The following pictures show the expression of MBNL1, and PTBP1:

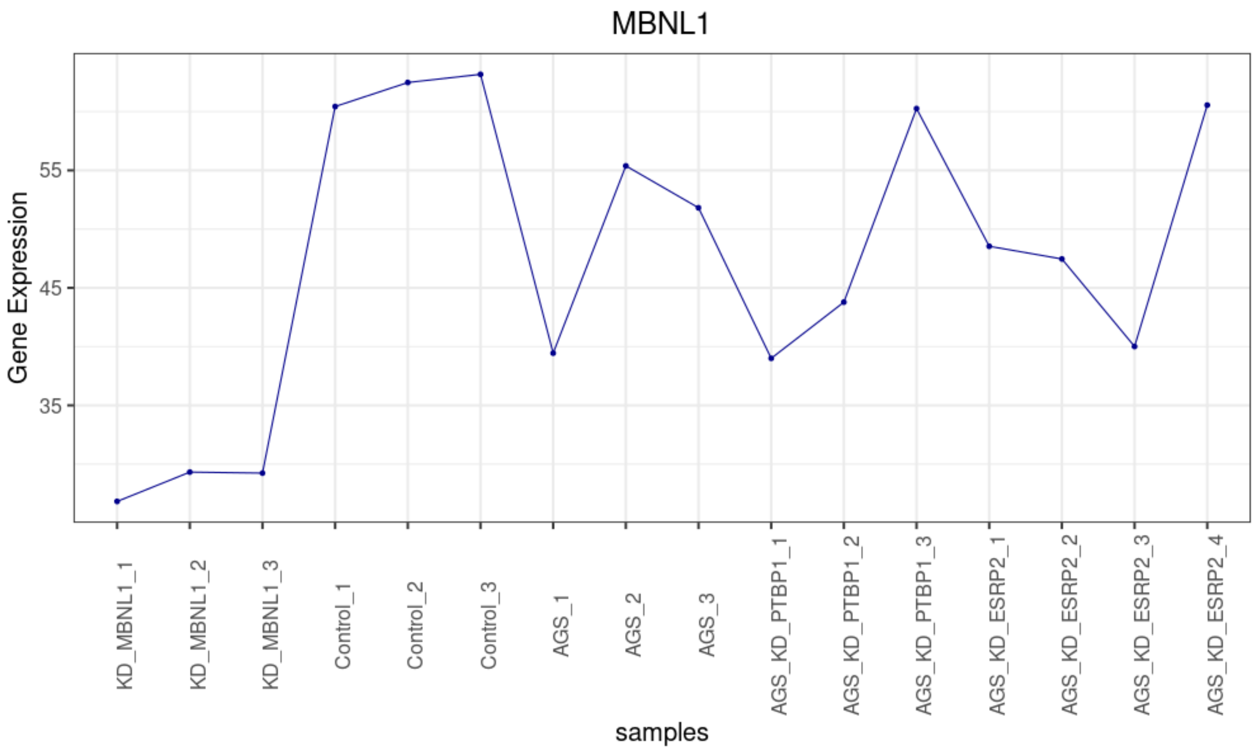

Figure S5. Expression of the MBNL1 gene throughout the samples of the experiment PRJEB39343. The first three samples correspond to the samples in which MBNL1 was knocked down.

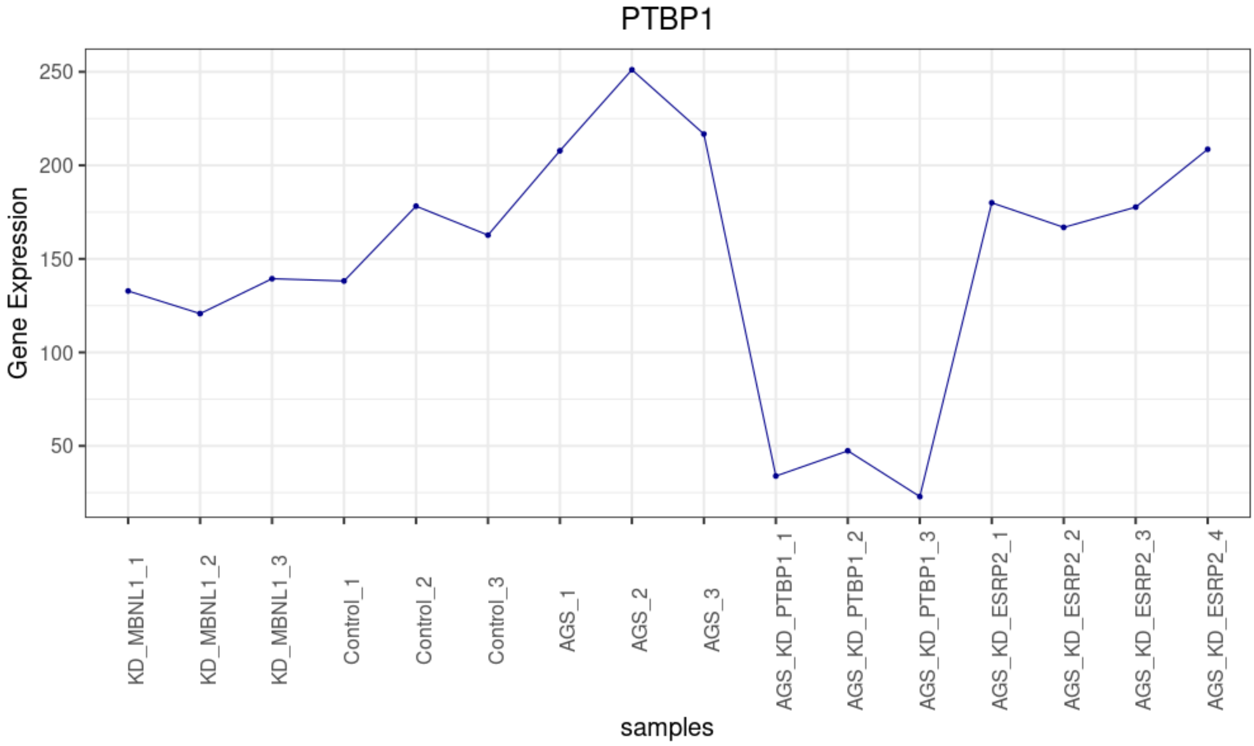

Figure S6. Expression of the PTBP1 gene throughout the samples of the experiment PRJEB39343. The tenth, the eleventh, and the twelfth samples correspond to the samples in which PTBP1 was knocked down.

GSE136366

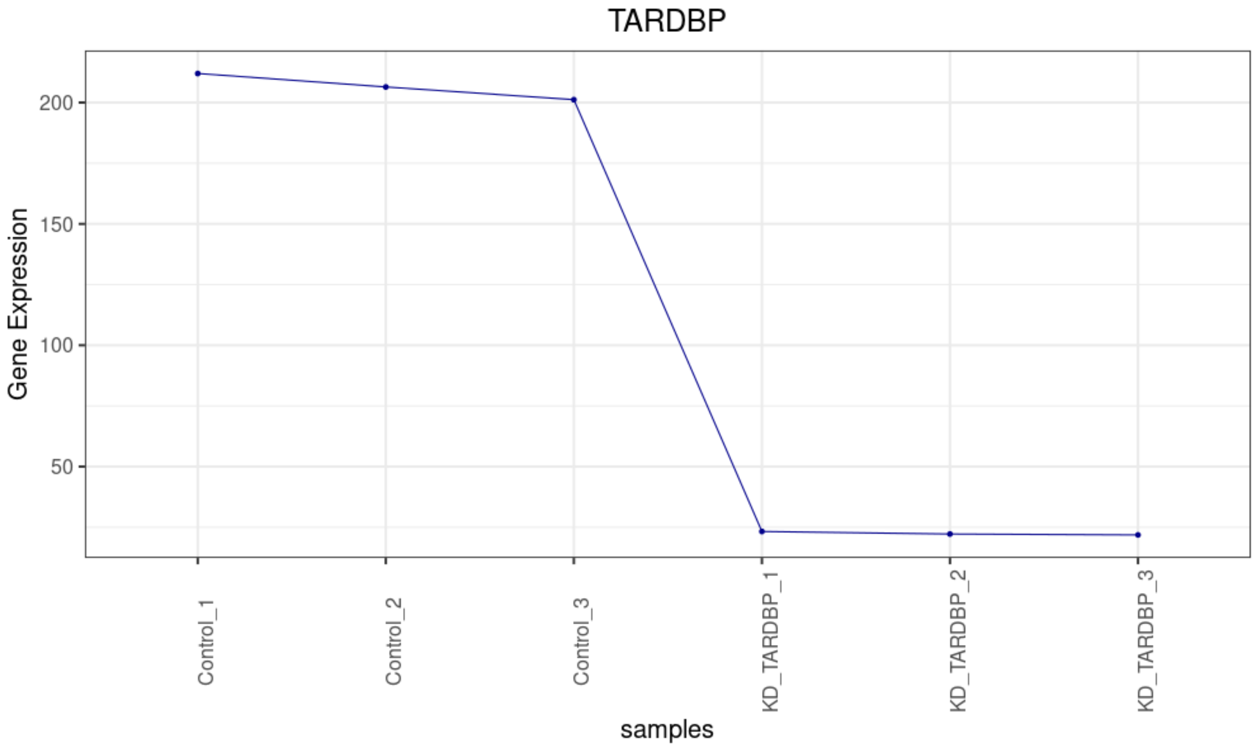

Figure S7. Expression of the TARDBP gene throughout the samples of the experiment GSE136366. The last three samples correspond to the samples in which TARDBP was knocked down.

GSE75491

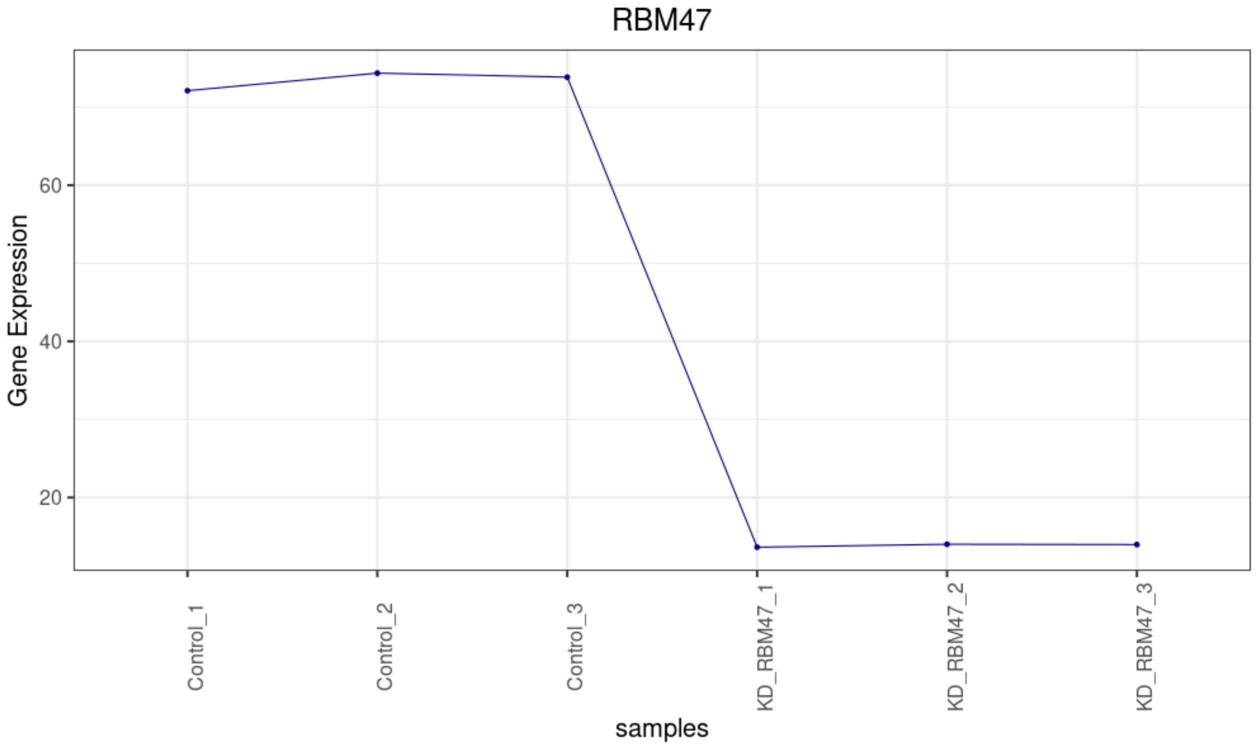

Figure S8. Expression of the RBM47 gene throughout the samples of the experiment GSE75491. The last three samples correspond to the samples in which RBM47was knocked down.

3 ENCODE Analysis

The ENCODE dataset includes 212 experiments corresponding to the knockdown of 106 different RBPs in HEGP2 and K562 cell lines. Most experiments feature 2 control and 2 knockdown samples with shared control samples used across multiple experiments. HEPG2 experiments utilized 21 distinct types of control sample sets, while K562 experiments employed 29 types.

Regarding the knockdown experiments, we found that the kncockdown of a targeted RBP also affects the expression of others RBPS, suggesting that there is an intricate relationship between the different RBPs. In fact, in all instances of the HEPG2 experiments, more than one RBP show exhibited differential expression (p.value < 0.001). We even found that the most under-expressed RBP was not the same as the RBP that was knocked out in 56 of the 106 cases. In 94 of the 106 experiments using the K562 cell line, more than one RBP showed differential expression, with the most under-expressed RBP not being the targeted RBP in 38 cases.

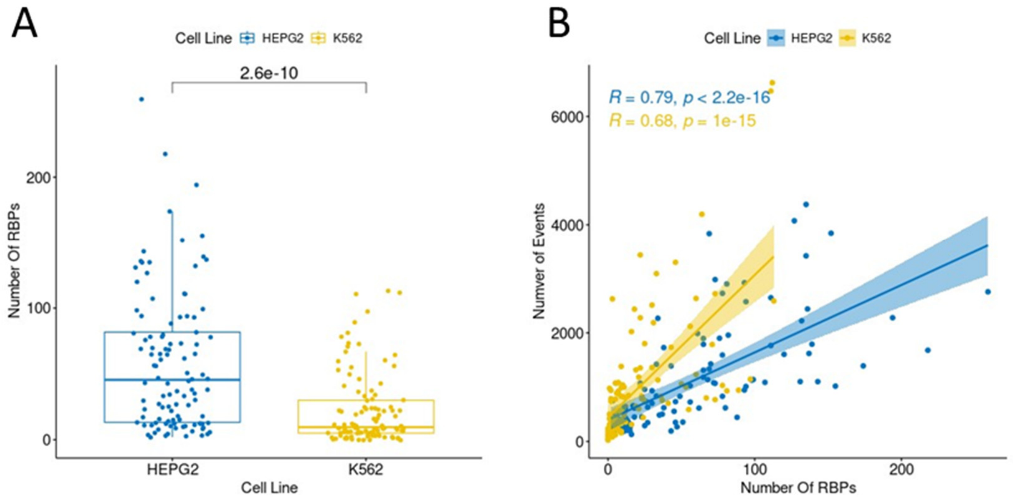

Figure S9. A) Number of RBPs differentially expressed in each knockdown experiment for HEPG2 and K562 cell lines. B) Correlation between the number of RBPs differentially expressed and the number of statistically significant splicing events.

A comparison of the RBP expression profiles between HEPG2 and K562 revealed a higher number of differentially expressed RBPs in HEPG2, as shown in Figure S9A. We also evaluated if the number of differential expressed RBPs affect the number of splicing events. As expected, we found a high correlation between the number of differentially expressed RBPs and splicing events (Figure S9B) with Spearman correlation coefficients of 0.79 for HEPG2 and 0.68 for K562. These findings imply that the function of an RNA binding protein (RBP) is interconnected with other RBPs, such that knocking down a single RBP can lead to splicing changes not directly attributed to the knocked-down RBP, but rather to other RBPs indirectly affected by the knockdown. Also, the results suggests that the consequences of a RBP knock-down depends on the cell type.

In addition, we examined the correlation of the $\Delta\Psi$ across the 106 experiments for each cell line. We observed that the experiments clustered together based on the control samples (Figure S10 and S12). The correlation between experiments with the same control is stronger than those with different

controls (Figures S11 and S13). This result is completely unexpected since using the ΔΨ should cancel out the effect of having the same reference, as the ΔΨ is a relative value. These results of the analysis suggest that the control samples of the experiment have more influence than the specific RBP that was knocked down.

HEPG2

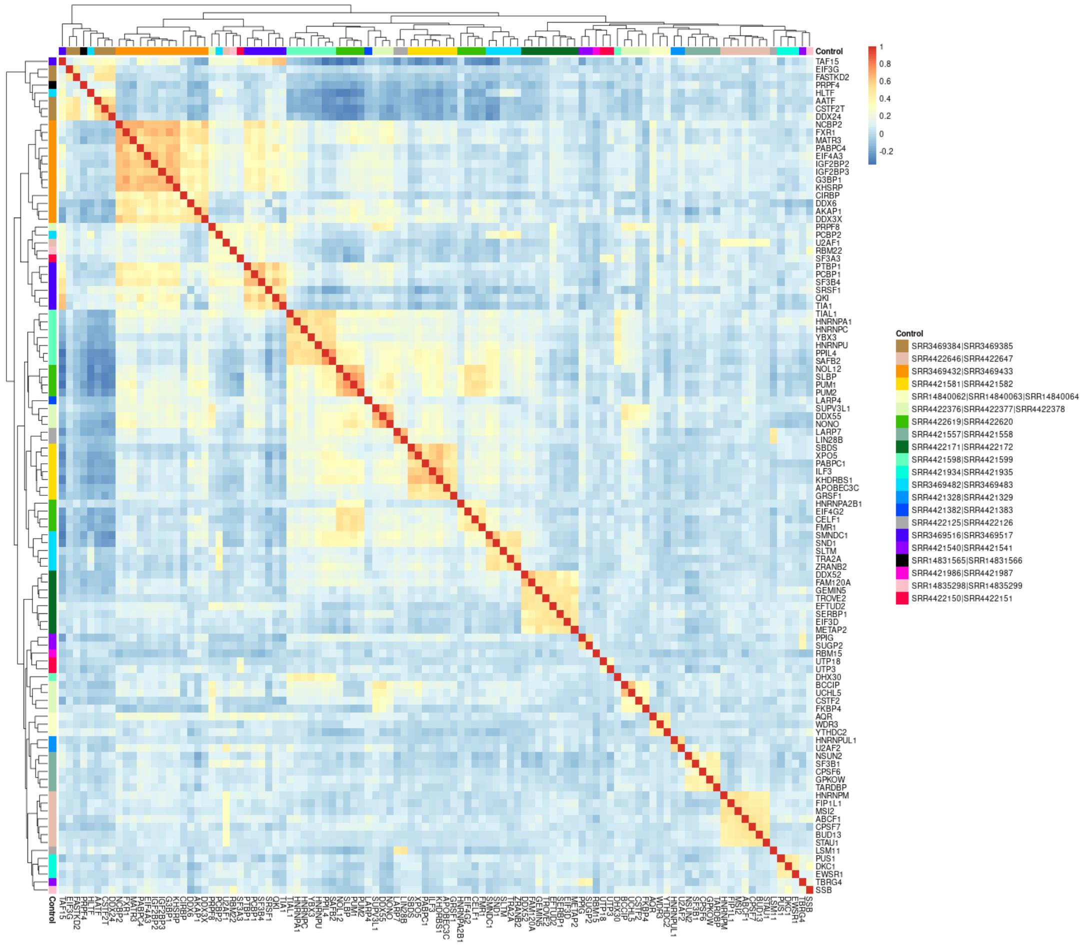

Figure S10. A heatmap illustrating the correlation of **ΔΨ** across HEPG2 experiments. The colors indicated along the top and left borders of the graph represent the respective sets of control samples for each experiment. It can be observed that the experiments tend to cluster according to their control sample sets.

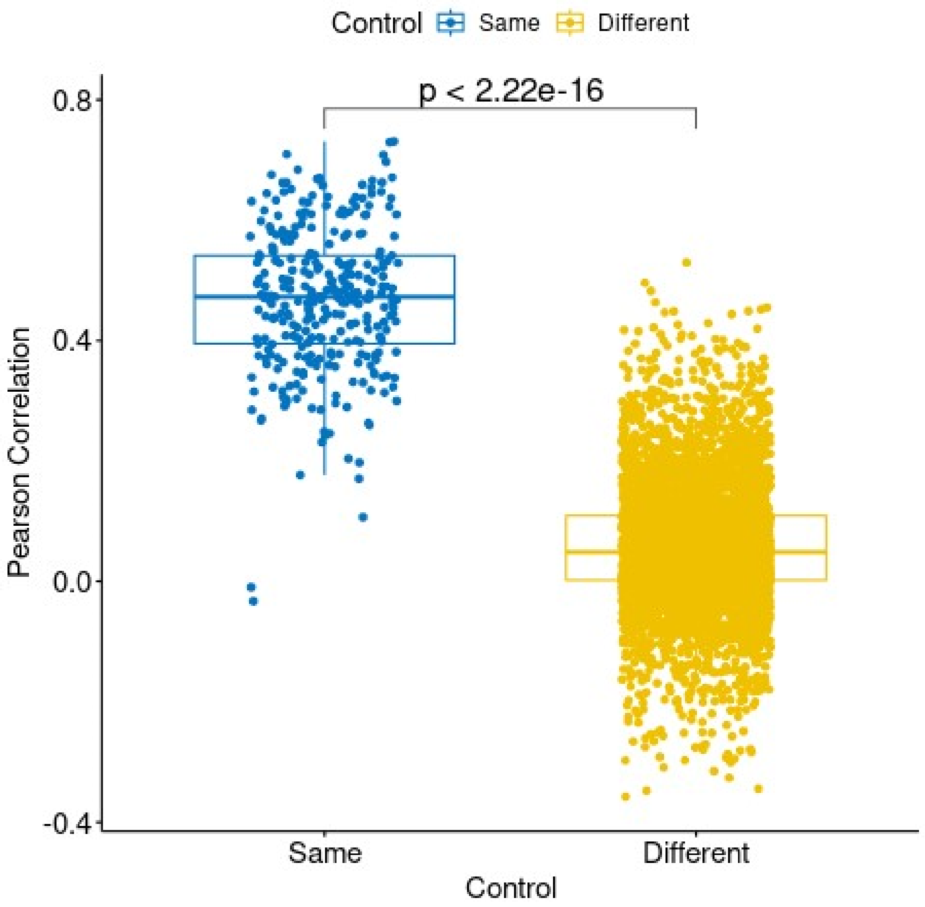

Figure S11. Pearson correlation of the ΔΨ of the samples with the same control samples in blue and with different control samples in yellow.

K562

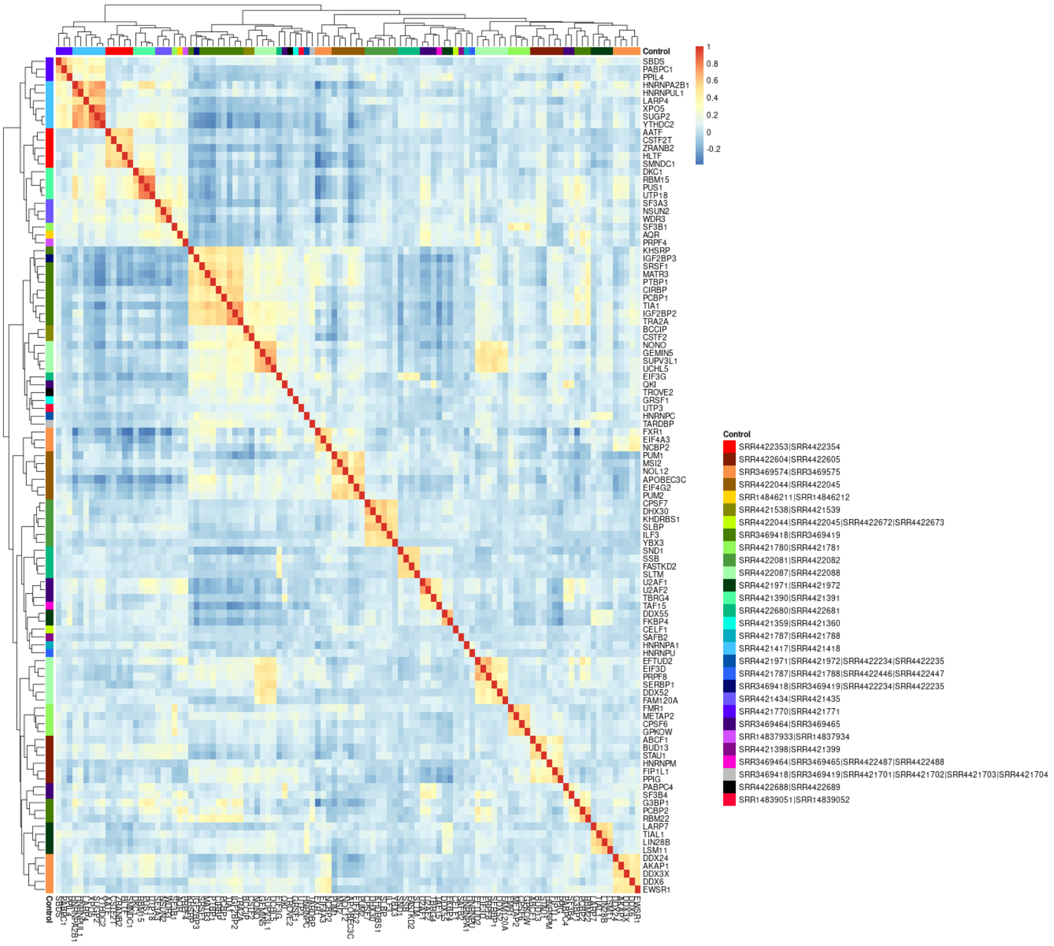

Figure S12. A heatmap illustrating the correlation of ΔΨ across K562 experiments. The colors indicated along the top and left borders of the graph represent the respective sets of control samples for each experiment. It can be observed that the experiments tend to cluster according to their control sample sets.

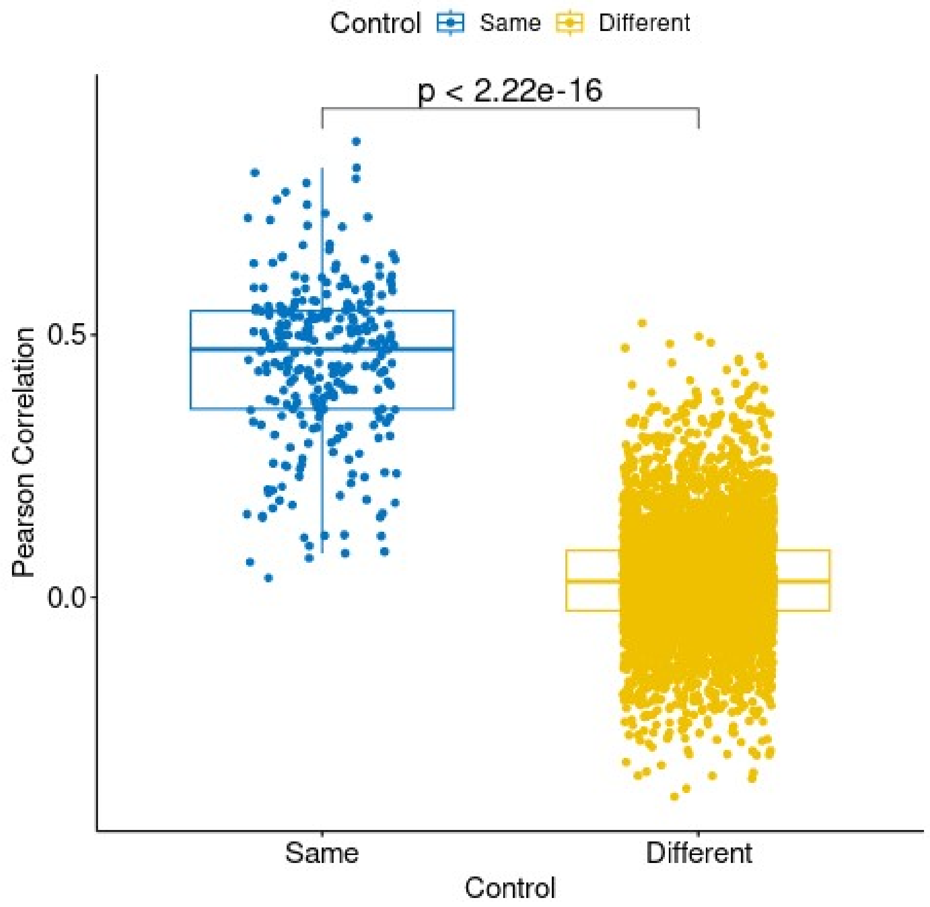

Figure S13. Pearson correlation of the ΔΨ of the samples with the same control samples in blue and with different control samples in yellow.
